# First-in-human *in vivo* non-invasive assessment of intra-tumoral metabolic heterogeneity in renal cell carcinoma

**DOI:** 10.1259/bjrcr.20190003

**Published:** 2019-03-04

**Authors:** Maxine Tran, Arash Latifoltojar, Joana B. Neves, Marianthi-Vasiliki Papoutsaki, Fiona Gong, Arnaud Comment, Ana S. H. Costa, Matthias Glaser, My-Anh Tran-Dang, Soha El Sheikh, Wivijin Piga, Alan Bainbridge, Anna Barnes, Tim Young, Hassan Jeraj, Ramla Awais, Sola Adeleke, Christopher Holt, James O’Callaghan, Frazer Twyman, David Atkinson, Christian Frezza, Erik Årstad, David Gadian, Mark Emberton, Shonit Punwani

**Affiliations:** 1 Research Department of Surgical Biotechnology, Division of Surgery & Interventional Science, Faculty of Medical Sciences, University College London, London, UK; 2 Specialist Centre for Kidney Cancer, Royal Free London NHS Foundation Trust, London, UK; 3 Centre for Medical Imaging, Division of Medicine, University College London, UK; 4 General Electric Healthcare, Chalfont St Giles, UK; 5 Medical Research Council Cancer Unit, Hutchison/MRC Research Centre, University of Cambridge, UK; 6 Institute of Nuclear Medicine, University College London Hospitals NHS Foundation Trust, London, UK; 7 Department of Chemistry, University College London, UK; 8 Department of Histopathology, Royal Free London NHS Foundation Trust, London, UK; 9 Department of Medical Physics and Biomedical Engineering, University College London Hospitals NHS Foundation Trust, London, UK; 10 Pharmacy Department, University College London Hospitals NHS Foundation Trust, London, UK; 11 Institute of Child Health, University College London, London, UK; 12 Faculty of Medical Sciences, School of Life and Medical Sciences, University College London, UK; 13 Department of Urology, University College London Hospitals NHS Foundation Trust, London, UK; 14 Department of Radiology, University College London Hospitals NHS Foundation Trust, London, UK

## Abstract

Intratumoral genetic heterogeneity and the role of metabolic reprogramming in renal cell carcinoma have been extensively documented. However, the distribution of these metabolic changes within the tissue has not been explored. We report on the first-in-human *in vivo* non-invasive metabolic interrogation of renal cell carcinoma using hyperpolarized carbon-13 (^13^C) MRI and describe the validation of *in vivo* lactate metabolic heterogeneity against multi regional *ex vivo* mass spectrometry. hyperpolarized carbon-13 (^13^C)-MRI provides an *in vivo* assessment of metabolism and provides a novel opportunity to safely and non-invasively assess cancer heterogeneity.

## Introduction

Intratumoral genetic heterogeneity in renal cell carcinoma (RCC) has provided important insights into the evolutionary pathway of RCC tumorigenesis.^1^ However, routine analysis of genetic intratumoral heterogeneity has yet to translate usefully into clinical practice as it requires specialized multiregional tumor sampling, complex computational analysis and sequencing platforms.

Metabolic reprogramming is a feature common to many solid tumors. Increased glucose uptake, glycolysis and reduced oxidative phosphorylation, known as the Warburg effect,^2^ have been reported in RCC.^3^ Hyperpolarized carbon-13 (^13^C) MRI (HP-MRI) is a novel non-ionizing imaging technique that allows non-invasive real-time analysis of metabolic pathways *in vivo*.^4^ Hyperpolarization using dissolution-dynamic nuclear polarization (DNP) technology provides unprecedented sensitivity for the detection of metabolism of ^13^C-labeled substrates such as pyruvate, fumarate and glucose *in vivo*.^4^ For example, following administration of 1-[^13^C] pyruvate, a number of studies have reported on the detection of 1-[^13^C] lactate via the reaction catalyzed by lactate dehydrogenase (LDH). This technique has recently been successfully translated into the clinical domain and is a promising tool for disease characterization and therapeutic response monitoring in prostate and brain tumors.^5–8^

Here, we report on the first-in-human non-invasive metabolic interrogation of RCC using HP-MRI and describe the validation of *in vivo* lactate metabolic heterogeneity imaged using HP-MRI against multiregional *ex vivo* mass spectrometry.

## Methods and materials

**Figure 1.**
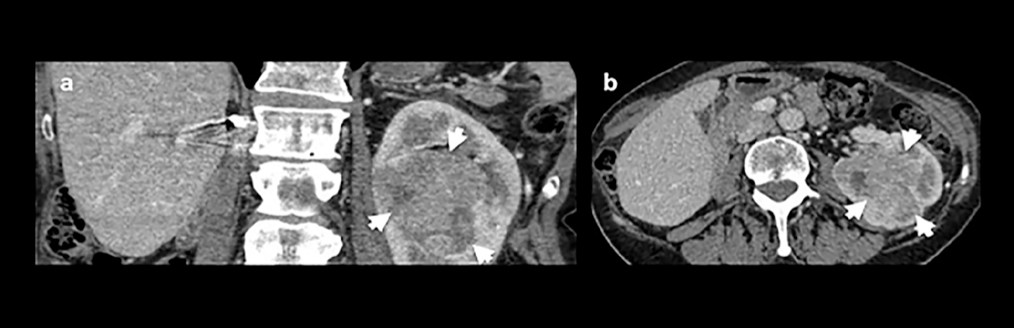
Contrast-enhanced CT of the abdomen at the level of left kidney. (a) Coronal and (b) axial slices showing an incidental finding of a 6.8 × 5.1 × 6.1 cm mass in the left kidney (arrows). There were no radiological signs of metastatic disease on CT of chest, abdomen and pelvis.

A 72-year-old female with a history of a previous right radical nephrectomy (17 years earlier) for clear cell RCC had an incidental finding of a 6.8 × 5.1 × 6.1 cm mass on the left kidney (Figure 1) confirmed as clear cell RCC.

Histopathological analysis of laparoscopic radical nephrectomy specimen confirmed ISUP/WHO Grade two clear cell RCC (staging pT3a).

The patient provided written informed consent for HP-MRI (Research Ethics Committee (REC) reference number 17/LO/0431) (ClinicalTrials.gov Identifier: NCT03687645 ) and for tissue based assays (REC reference number 16/WS/0039).

### Production of hyperpolarized 1-[^13^C] pyruvate

Hyperpolarized 1-[^13^C] pyruvate solution was filled and assembled under aseptic conditions then produced using a DNP polarizer (SPINlab, GE Healthcare, Milwaukee, Wisconsin) and a sterilized fluid path.^9^ The filled sterilized fluid path was loaded into the hyperpolarizer and the sample, consisting of 1.47 g 1-[^13^C] pyruvic acid (GMP Precursor from Sigma Aldrich, Vienna, Austria) doped with 15 mM AH111501 electron paramagnetic agent, underwent microwave irradiation for approximately 2 h to achieve a polarization of 31.9%. The sample was then dissolved in 38 ml of sterile water and neutralized with 17.5 g sterile trometamol buffer solution (333 mM Tris and 600 mM NaOH) in 19 ml of sterile water. Release criteria for sterile hyperpolarized solution for injection are tabulated in Table 1.

**Table 1.** Release criteria for final product

**Release criteria for sterile hyperpolarized solution in Medrad syringe for injection**	
Appearance	Clear colorless solution with a slightly green tinge and free from visible particles
Validations satisfactory for	Sterility: Complies with Ph. Eur. Endotoxins: Complies with Ph.Eur.
Physical & chemical parameters	Based on UCSF Limits
^13^C nuclear polarization	Not Less Than 10.0%^a^
Pyruvate	220–280 mM
Residual AH111501	Not more than 3.0 µM
pH (i) QC module (ii)pH strips ∆ (i) & (ii)	6.5–8.5 6.5–8.5 ≤1.0 pH unit
Drug product temperature	25.0 – 37.0^o^ C^b^
Drug product volume	>38 ml
Compliance with TSE regulations	

TSE, transmissible spongiform encephalopathies.

a Polarization at the start of dissolution. UCSF limit NLT 15%

b Temperature at the time of analysis

### Imaging set-up

The patient was positioned in a clinical 3 T integrated PET-MR scanner (Siemens Biograph mMR, Enlargen, Germany) in the supine position with an intravenous (i.v.) catheter placed in the left arm. A specialized custom-design clamshell ^13^C transmit coil was used with two (anterior and posterior) 7-channel ^1^H/^13^C receive surface phase array coils (RAPID Biomedical GmbH, Rimpar, Germany) for signal excitation and reception of ^13^C signals respectively.

The i.v. line was connected to an automatic dual chamber power injector with the first chamber (chamber A) programmed to deliver hyperpolarized 1-[^13^C] pyruvate solution at a rate of 5 ml s^−1^. The second chamber (chamber B) was pre-loaded and programmed to deliver 20 ml of normal saline flush immediately after hyperpolarized solution injection.

Anatomical localization of the renal tumor was performed on axial and coronal *T*_2_ weighted imaging. A turbo spin echo sequence was utilized with the following parameters: repetition time = 5400 ms, effective echo time = 111 ms, slice thickness = 3 mm, number of slices = 30, Field of view (axial) = 203 mm x 460 mm, field of view (coronal) = 369 mm x 460 mm, echo train length = 15, number of signal averaging = 1.

Following localization of the tumor, a central axial imaging slice was planned under the direction of a board certified radiologist for subsequent ^13^C imaging.

### Hyperpolarized MRI

40 ml of hyperpolarized 1-[^13^C] pyruvate was injected at a rate of 5 ml s^−1^ followed by a flush of 20 ml of normal saline at 3 ml s^−1^. Repeated ^13^C chemical shift imaging (CSI) measurements were performed (repetition time = 80 ms, time of echo = 3 ms, flip angle = 10°, bandwidth = 10,000 Hz, field of view = 120 mm x 120 mm, slice thickness = 30 mm, acquisition matrix = 16 × 16), commencing at a delay of 25 s following start of injection to allow for sufficient hyperpolarized solution delivery to the kidney. Single-slice CSI images were acquired every 20 s for a total of 12 repetitions.

The CSI data were analyzed offline (MATLAB 2016; MathWorks Inc., Natick, MA). The individual free induction decays across the CSI grid were apodized with an exponential 10 Hz filter in the time domain and then Fourier transformed. The areas of spectral peaks were calculated to produce the metabolic maps. Metabolic maps of the 1-[^13^C] labeled lactate and pyruvate spectral areas and lactate/pyruvate ratio were generated for the first acquired CSI dataset (demonstrating the highest formation of 1-[^13^C] lactate). The 1-[^13^C] lactate signal was not measurable on subsequent CSI data.

### Tissue handling

After macroscopic pathology review, multiregional tissue samples were collected within 30 min of nephrectomy. A 1 cm thick axial slice of kidney was selected at the level of the renal hilum (visually matched to the MRI CSI imaging slice) and regional sampling was labelled sequentially (10 samples of tumor, and 5 samples of adjacent non-tumorous kidney tissue). Samples were placed in cryo-vials and immediately snap frozen with liquid nitrogen. Samples were formalin fixed, processed in paraffin and stained with hematoxylin & eosin after sectioning for microscopic confirmation of presence or absence of malignancy.

### Lactate measurement by liquid chromatography-mass spectrometry

Frozen tissue samples were weighed into Precellys tubes prefilled with ceramic beads (Stretton Scientific Ltd., Derbyshire, UK), and an exact volume of extraction solution (30% acetonitrile, 50% methanol and 20% water) was added to obtain 40 mg specimen per mL of extraction solution. Samples were lyzed using a Precellys^®^24 tissue homogeniser (Bertin Corp, Rockville, MD. 5500 rpm 15 s x 2) and then centrifuged (16,162 x g for 10 min at 4°C). The supernatant was transferred to glass vials (Microsolv Technology Corp., Leland, NC) and stored at −80°C until LC-MS analysis.

Samples were randomized in order to avoid bias due to machine drift and the operator was blind to the HP-MRI assessment. LC-MS analyses were performed on a Q Exactive mass spectrometer (Thermo Fisher Scientific) mass spectrometer coupled to an Ultimate 3000 RSLC system (Dionex). The liquid chromatography system was fitted a ZIC-pHILIC column (150 × 2.1 mm) and respective guard (20 × 2.1 mm) (all Merck Millipore, Germany), and metabolites were eluted with the previously described gradient.^10^ The mass spectrometer was operated in full MS and polarity switching mode. The acquired spectra were analyzed using XCalibur Quan Browser software (Thermo Fisher Scientific). Absolute quantification of lactate was performed by interpolation of the corresponding standard curve obtained from serial dilutions of a commercially available standard (Sigma Aldrich, Vienna, Austria) running with the same batch of samples.

## Results

1-[^13^C] Lactate signal was observed on the first, and 1-[^13^C] pyruvate signal on the first and second acquisition of the CSI temporal series.

**Figure 2.**
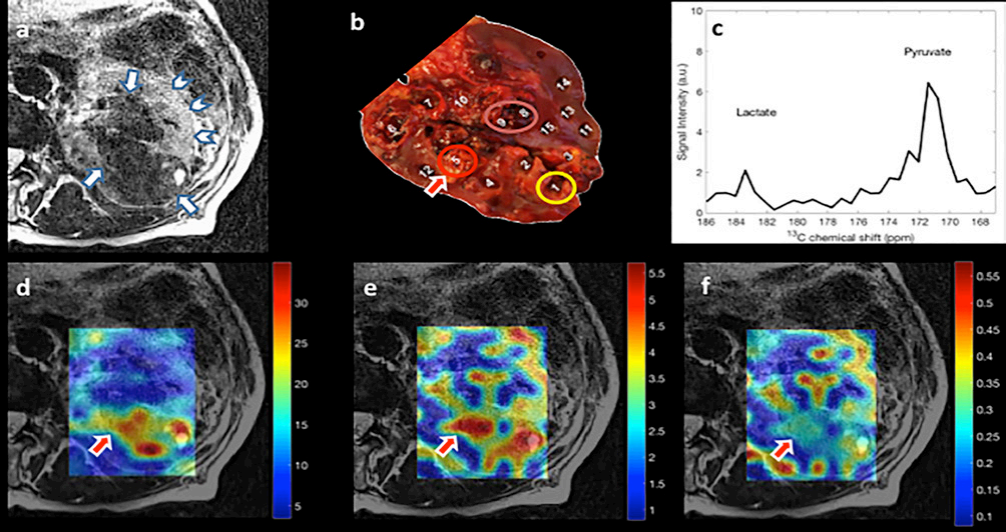
(a) Axial *T_2_* weighted turbo spin echo MRI through kidney. Renal tumor (arrows) and normal-appearing renal tissue (arrow head) are shown. (b) 1 cm thick axial slice of kidney at the level of the renal hilum and 15 tissue samples (1–10 tumor samples and 11–15 non-tumor samples). Images from ^13^C CSI first acquisition (c–f). (c) Corresponding spectra of the arrowed region on (b) (region 5). The spectrum shows prominent signals from lactate (left) and pyruvate (right). (d) Interpolated hyperpolarized 1-[^13^C] pyruvate map overlaid on *T_2_* weighted turbo spin echo MRI. (e) Interpolated hyperpolarized 1-[^13^C] lactate map overlaid on *T_2_* weighted turbo spin echo MRI. (f) Interpolated hyperpolarized 1-[^13^C] lactate/pyruvate map overlaid on *T_2_* weighted turbo spin echo MRI. Bottom row depicts intratumoral heterogeneity of; pyruvate delivery (d), pyruvate to lactate metabolic conversion (e) and the ratio of lactate to pyruvate map (f) (a.u.). CSI, chemical shift imaging.

The 1-[^13^C] pyruvate signal (Figure 2d) provides a measure of blood flow, whilst the 1-[^13^C] lactate signal demonstrates metabolism (Figure 2e).^9^

Heterogeneous 1-[^13^C] pyruvate signal was seen across the RCC, reflective of variability of blood flow (Figure 2d). Heterogeneous formation of 1-[^13^C] lactate is also evident (Figure 2e). Moreover, areas of high 1-[^13^C] lactate formation did not always conform to areas of high blood flow as highlighted by the lactate/pyruvate ratio map (Figure 2f).

**Figure 3.**
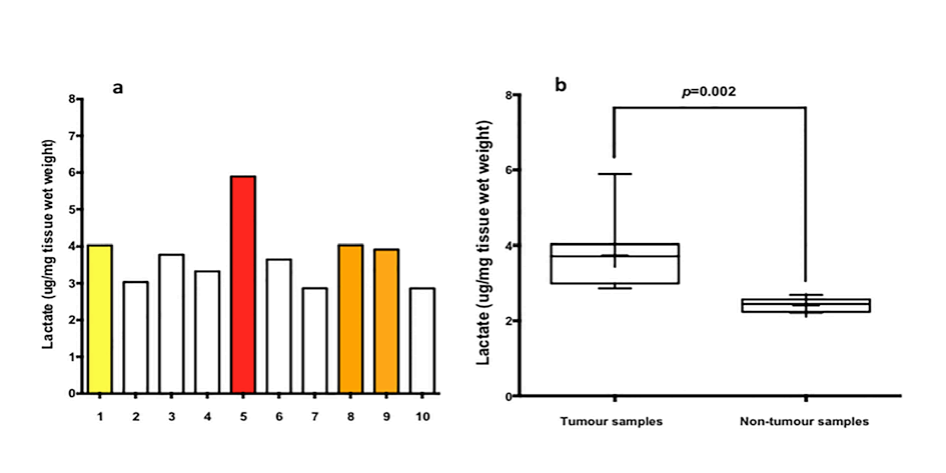
(a) Bar chart of liquid chromatography–mass spectrometry analysis. The graph depicts liquid chromatography–mass spectrometry analysis of the multiregional samples from the tumor. The highest level of lactate accumulation was found in region 5 (red bar) in consistence with the findings of hyperpolarized 1-[^13^C] lactate map (Figure 2e, red arrow). Colored bars correspond to colored circles in Figure 2b. (b) Box and whisker plot of liquid chromatography–mass spectrometry analysis of the multiregional samples from tumor and non-tumor areas. The median and interquartile range of the weight of lactate per sampled area was 3.7 μg/mg of tissue wet weight and 0.89 for tumor samples and 2.4 μg/mg of tissue wet weight and 0.18 for non-tumor samples respectively (*p* = 0.002). The boundaries of the box show 25th and 75th percentiles, and the line within the box is the median. Whiskers show 10th and 90th percentiles.

LC-MS analysis confirmed heterogeneous lactate levels within tumor samples (Figure 2b, samples 1–10), as depicted on the bar chart in Figure 3. The highest level of lactate by mass spectrometry (Figure 3—red bar) was found in region 5, corresponding to the region displaying the highest 1-[^13^C] lactate signal in the HP-MRI scan (Figure 2—red arrow). Further 1-[^13^C] lactate formation was observed at imaging corresponding to samples 8–9 (Figure 3a—orange bars) and sample 1 (Figure 3a—yellow bar).

Overall, we observed heterogeneity of HP-MRI 1-[^13^C] lactate signal generally conforming to the heterogeneity found at mass spectrometry metabolic analysis.

## Discussion and conclusion

In this study, we report on the first-in-human *in vivo* non-invasive metabolic assessment of RCC using 1-[^13^C] pyruvate HP-MRI and validate the results with *ex vivo* multi regional LC-MS analysis.

HP-MRI using dissolution DNP produces a dramatic signal enhancement, allowing real-time imaging of metabolic pathways.^4^ Until now, the clinical application of HP-MRI in malignancies has been limited to prostate cancer^5,6^ and brain tumors.^7,8^ To the best of our knowledge this study represents two novel factors: it is the first-in-human 1-[^13^C] pyruvate HP-MRI study in RCC, and it is the first HP-MRI study with tissue assay validation.

Unlike the multiregional sampling used for tissue-based assessment of genetic and metabolic heterogeneity, HP-MRI provides a non-invasive technique that can be repeated over time and thus could be used for longitudinal tumor monitoring.^6^

It has been previously shown that the pyruvate signal build-up precedes the lactate signal build-up in the time course data of pre-clinical animal models, consistent with the notion that the pyruvate signal reflects its delivery to the tissue and cells whilst the lactate signal reflects pyruvate-to-lactate conversion catalyzed by lactate dehydrogenase in the glycolytic pathway.^11^

In our patient, the regional distribution of pyruvate and lactate differed markedly across the RCC, suggesting that blood flow and metabolism were not integrally linked within the cancer.

One possible explanation for our observation is that higher levels of LDH are found in areas of tumor which exhibit hypoxia.^12^ Tumor hypoxia can be caused by high levels of metabolic activity or through relative decrease in effective blood flow.^13^ Hence low 1-[^13^C] pyruvate signal, reflecting limited blood flow, may occur together with high 1-[^13^C] lactate signal, reflecting increased metabolic activity.

We also observed heterogeneity of *in vivo* 1-[^13^C] lactate signal across the tumor confirmed by *ex vivo* mass spectrometry. Genomic, microstructural and macrostructural heterogeneity has been noted in cancers including RCC.^1,14,15^ Our results confirm heterogeneous metabolic activity is also present within RCC. Future work linking genomic, metabolic, microstructural and macrostructural changes is needed to explore if RCC metabolic heterogeneity relates to genomic heterogeneity; and, whether metabolic heterogeneity is a result of micro/macrostructural heterogeneity or conversely results in RCC micro/macrostructural heterogeneity.

The CSI sequence used within this study enabled single slice imaging with limited temporal resolution. New sequences are being developed for hyperpolarized ^13^C metabolic imaging allowing full anatomical coverage and improved temporal resolution.^16–18^

HP-MRI has a variety of potential clinical applications. For example, knowledge of RCC metabolic activity may provide a prognostic tool to help treatment stratification, such as whether active surveillance or urgent surgery is warranted. Temporal changes in metabolism could be used to monitor patients on active surveillance, and to provide an assessment of treatment response. Indeed, identifying areas of higher metabolic activity within solid tumors in general could help guide target biopsy and themselves act as targets for novel focal therapies.

### Learning points

1. Metabolic reprogramming is a feature common to many solid tumors with increased glucose uptake, glycolysis and reduced oxidative phosphorylation (Warburg effect) reported in RCC
2. Hyperpolarized carbon-13 (^13^C) MRI is a novel non-ionizing imaging technique that allows non-invasive real-time analysis of metabolic pathways *in vivo.*
3. We report on the first-in-human *in vivo* non-invasive metabolic assessment of RCC using 1-[^13^C] pyruvate HP-MRI and validate the results with *ex vivo* multiregional liquid chromatography–mass spectrometry analysis.
4. The regional distribution of pyruvate and lactate differed markedly across the RCC, suggesting that blood flow and metabolism were not integrally linked within the cancer
5. Knowledge of RCC metabolic activity using hyperpolarized carbon-13 (^13^C) MRI may provide a future prognostic tool to help treatment stratification.
